# Hydrothermal Pretreatment Plus Supercritical CO_2_ Foaming as a Novel Route to Improving Polymer Structures for Biomedical Applications—Part 1: Preliminary Screening for Individual and Combined Polymers

**DOI:** 10.3390/polym18010081

**Published:** 2025-12-27

**Authors:** M. Belén García-Jarana, Ramón Terroba, José M. Vázquez-Fernández, Diego Valor, Clara Pereyra, Juan R. Portela

**Affiliations:** Department of Chemical Engineering and Food Technology, Faculty of Sciences, Wine and Agrifood Research Institute (IVAGRO), University of Cadiz, 11510 Cadiz, Spain; ramon.terrobaparrado@alum.uca.es (R.T.); josemanuel.vazquez@uca.es (J.M.V.-F.); diego.valor@uca.es (D.V.); clara.pereyra@uca.es (C.P.); juanramon.portela@uca.es (J.R.P.)

**Keywords:** scaffolds, hydrothermal, pretreatment, polymers, CO_2_, foaming

## Abstract

Degradable polymers are essential in tissue engineering due to their capacity to mimic the extracellular matrix and promote regeneration. To be functional, they require interconnected porous structures that allow for nutrient exchange and cell migration. Although methods exist to optimize porosity, many compromise biocompatibility because pore-forming substances are used. In this context, hydrothermal pretreatment emerges as a promising technique to simultaneously improve both the porosity and mechanical properties of polymers without using potentially toxic reagents. This study proposes a novel route that combines hydrothermal pretreatment with supercritical CO_2_ foaming, evaluating whether the structures obtained present better characteristics for biomedical applications compared to those obtained using supercritical CO_2_ foaming alone. A screening of this novel route has been tested on individual polymers (PCL, PLA, PLGA, PVA, PBS, chitosan) and various binary combinations (PCL-PBS, chitosan-PBS, PVA-PBS, PLGA-PEDOT: PSS). The resulting materials were characterized using electron microscopy to analyze pore diameter and distribution, as well as structural stability and homogeneity. For the individual polymers, the hydrothermal pretreatment clearly improved the results obtained. However, most polymer combinations showed drawbacks such as mass losses, heterogeneity, or unsatisfactory pore formation. This research highlights the potential of hydrothermal pretreatment to optimize scaffolds, which is crucial for viability in biomedical applications.

## 1. Introduction

Biodegradable polymers have emerged as a sustainable solution to plastic pollution, offering a viable alternative to conventional polymers that remain in the environment for decades. Their ability to degrade under specific conditions has attracted significant interest in various industries. Materials such as polylactic acid (PLA), polyhydroxyalkanoates (PHA), polycaprolactone (PCL), and polybutylene succinate (PBS) have demonstrated promising properties that allow for their use in a range of applications that go from food packaging to biomedical devices. In particular, PLA, which is derived from renewable sources such as corn, has gained popularity based on its controllable biodegradability and low production cost. PHAs, produced by microorganisms, stand out due to their biocompatibility and versatility in medical applications, while PCL and PBS exhibit well-balanced mechanical properties that render them suitable for both medical devices as well as for packaging [1,2]. However, these polymers still face certain challenges in terms of implementation, such as the need to optimize their degradation process or improve their mechanical properties so that they can compete against traditional polymers [3]. In the biomedical field, biodegradable polymers have gained considerable relevance in the development of medical devices, drug delivery systems, and scaffolds for tissue engineering. Their biocompatibility makes them suitable for implantation, while their biodegradability ensures they do not generate long-term residues in the body. This combination makes them an interesting option for cell regeneration and regenerative medicine. PCL, for example, has been extensively tested to produce bone and cartilage tissue-regenerating scaffolds. In addition, certain advances in the techniques for the modification of these polymers have improved their stability and functionality, thereby unlocking new potential applications in the healthcare sector. However, their degradation rate varies depending on environmental conditions, and their mechanical resistance still represents a challenge in certain applications, which demand further research on new formulae and processes to optimize their performance [4]. Polymers play an essential role in biomedicine, as their capacity to suit a variety of medical applications allows their usage in tissue engineering, controlled drug release, or the development of advanced medical devices. Thanks to their versatility, they can be used to manufacture three-dimensional scaffolds that promote the regeneration of cells from different tissues, such as bone, skin, or cartilage, and for the precise dosing of drugs through controlled-release systems. They are also essential in biomedical implants, including orthopedic prostheses, biocompatible coatings, and resorbable sutures, as they enhance the integration of these implants into the body and have a reduced risk of rejection. They also excel in the development of hydrogels for regenerative medicine and in the 3D printing of tissues and polymeric nanoparticles, which opens up new possibilities for customized therapies and advanced treatments [5,6,7]. As materials intended for clinical use, biomedical polymers are inherently biocompatible. Many of them also exhibit biodegradable behavior, which reduces the risk of adverse patient reactions and facilitates safe resorption or elimination once they have fulfilled their function. Their properties, such as porosity, cell adhesion, or degradation rate, can be adjusted so that they become more suitable for tissue regeneration processes. Furthermore, they can be designed to mimic the mechanical and thermal resistance of the original tissue in order to provide temporary support while the tissue fully regenerates [8]. The combination of polymers can be very useful for improving the multiple properties of polymers. For example, PBS is a key biopolymer valued for its melt processability and inherent biodegradability, but its application is constrained by low mechanical strength. Thus, its blend with PVA can address this shortcoming, also acting as a nucleating agent and improving rheological properties [9]. On the other hand, the blending of PBS and PCL offers a promising strategy to obtain materials with an optimized profile of physical and biodegradation properties. Individually, PCL exhibits a higher degradation rate but contributes superior tensile strength, while PBS, with a significantly slower degradation rate, provides enhanced toughness. Therefore, forming PBS/PCL blends may improve the balance of strength and toughness and make them better-suited for biomedicine applications [10]. Additionally, a mixture of a non-conductive polymer (PCL, PLA, PLGA, etc.) with a conductive one (PEDOT, etc.) has already been proven to play a key role in neural implants, as it allows for the transmission of electrical signals and improves the communication between cells in muscle-stimulation or nerve-repair processes. The HT treatment was primarily used to obtain a combined polymer via a fusion of both individual polymers, obtaining a single solid including both components. In certain fields where studies are more advanced, conductive polymers, such as polypyrrole and PEDOT: PSS, have already proven their capacity to play a key role in neural implants and implantable electronic devices, as they allow for the transmission of electrical signals and improve the communication between cells in muscle-stimulation or nerve-repair processes. These advances highlight the importance of a proper modification and design of the polymers for the development of innovative and customized biomedical solutions [11]. Table 1 lists the different biomedical applications based on each polymer’s properties.

Supercritical technologies have gained recognition for their ability to process materials under specific temperature and pressure conditions that modify the properties of fluids. Supercritical CO_2_, in particular, has been consolidated as a key solvent and expanding agent for the manufacturing of innovative polymeric materials on account of its non-toxic, non-flammable nature and its easy recovery [28]. When it surpasses its critical point (31.1 °C and 73.8 bar), it exhibits properties that are between those of a liquid and a gas. These properties make it an ideal agent for extractive, impregnating and synthesizing processes in areas such as tissue engineering. Its capacity to dissolve certain compounds makes it suitable for the development of the porous three-dimensional structures known as scaffolds, which are used to replicate the extracellular matrices and promote cell growth in biomedical applications [29].

The supercritical CO_2_ foaming process, which consists of dissolving the gas into the polymer under high-pressure conditions followed by rapid depressurization to form a porous matrix, is one of the most widely used methods to produce scaffolds [30]. This approach has been key in the production of biodegradable foams, such as polylactic acid (PLA) foams, which are used for controlled drug-releasing devices and for tissue regeneration [31]. Supercritical CO_2_ acts as a non-toxic blowing agent that facilitates pore nucleation and growth during depressurization by dissolving into the polymer matrix and plasticizing the material. Porosity management is achieved by carefully adjusting the saturation pressure and temperature, which determine the amount of CO_2_ uptake and the extent to which the polymer swells. Rapid and controlled depressurization is intended to promote a high and homogeneous nucleation rate, thereby fostering the formation of a more uniform porous structure. Furthermore, the interplay between polymer–CO_2_ affinity and diffusion kinetics modulates the final pore structure. Among its advantages are the absence of toxic residues and the possibility of adjusting the microstructure by varying certain parameters such as the temperature and depressurization rate. However, the precise control of pore size and distribution still remains a challenge [32]. A hydrothermal pretreatment has arisen as a promising alternative for the optimization of these processes, as it improves the formation of porous structures with no need to add pore-forming agents that need to be removed at a later stage.

For the hydrothermal treatment, water at a high temperature and under a high pressure are used to modify or decompose the material without the intervention of aggressive chemical agents. This method allows the reactions to take place under subcritical or supercritical water conditions, which makes it attractive for waste valorization, nanomaterial synthesis and polymer treatment. Water in a subcritical state (100–374 °C, <22 MPa) favors the hydrolysis of polar compounds, while, in a supercritical state (>374 °C, >22 MPa), it favors oxidation and hydrolysis reactions due to its ability to dissolve non-polar compounds [33,34].

These properties were leveraged in a previous study [35] in which the application of this hydrothermal process before and after the supercritical CO_2_ foaming process for polycaprolactone was evaluated. In that previous work, the application of a hydrothermal process after the supercritical CO_2_ foaming process was discarded based on unsatisfactory results, such as pore collapse, mechanical weakness, and lower interconnectivity, which rendered the scaffolds unsuitable for structural applications. Hot compressed water, with a lower dielectric constant, acts less like a polar solvent and more like an organic solvent, increasing its solubility and reactivity. The water molecules penetrate the polymer matrix and position themselves between the polymer chains, therefore disrupting the strong intermolecular attractive forces (such as hydrogen bonds or Van der Waals forces) between the polymer chains. That disruption increases the free volume within the polymer structure and lowers the glass transition temperature (T_g_) so the polymer chains can move more freely and easily at the same operating temperature, producing an enhancement of chain mobility [36]. The high temperature and presence of water can lead to some chain scissions, potentially leading to a decrease in crystallinity or a change in the melting point. Decreased crystallinity, enhanced chain mobility, and increased free volume lead to a higher diffusion coefficient for small molecules (such as CO_2_) within the polymer matrix. However, water becomes highly reactive, particularly when operating at higher temperatures, acting as a nucleophile or an acid/base catalyst, so the temperature should be controlled to avoid attacks of hydrolyzable functional groups within the polymer backbone, such as ester, amide, ether, and acetal linkages. The hydrothermal pretreatment of polymers has been shown to improve the porosity and homogeneity of the materials, as the ability of water to penetrate and swell polymeric matrices allows for a controlled expansion; additionally, by reducing the crystallinity in highly crystalline polymers, it facilitates the subsequent permeation of a blowing agent such as CO_2_. This approach has been proven to improve the formation of the porous structures that are essential for biomedical applications and for advanced materials engineering [35].

Consequently, this study investigated the effect of a hydrothermal pretreatment followed by the supercritical CO_2_ foaming of a variety of polymers, such as polycaprolactone (PCL), polylactic acid (PLA), polylactic acid glycolic acid (PLGA), polyvinyl acetate (PVA), polybutylene succinate (PBS), and chitosan. Furthermore, the viability of combined polymers, such as PLGA and PEDOT: PSS, PVA and PBS, PCL and PBS, and chitosan and PBS, was determined.

For this purpose, we conducted a first screening of the novel route for all those polymers to check whether the products obtained meet the basic requirements for stability, homogeneity, and improvement in pore formation. In this way, this study (part 1) only includes the analysis of pore diameter and distribution, as well as the homogeneity and expansion degree of the resulting scaffolds. Better results were obtained for individual polymers than for combined polymers, observing in the later severe disintegration or lack of homogeneity in most cases. Those polymers showing the best results (PCL, PLGA, and PVA) were selected for further studies of this novel route (part 2), under a wider set of experimental conditions and including a characterization of the products structure in more detail, to assess their suitability for biomedical applications.

## 2. Materials and Methods

### 2.1. Materials

The polymers were supplied by Sigma-Aldrich (Steinheim, Germany); PCL, PVA, PBS (average Mw of 45,000 g∙mol^−1^) were supplied in pellet form, while PLA and PLGA (50:50 and 75:25) were supplied in powder form, and Chitosan and PEDOT: PSS were in liquid form.

For those polymers in powder form, 30 mg of sample was pressed into cylindrical pellets (approx. 7.6 mm diameter × 1 mm height) using a Qwik Handi Press (P/N 0016 125; Thermo Spectra Tech, Shelton, CT, USA) and a 7 mm Die Set (P/N 0016 111; Thermo Spectra Tech, Shelton, CT, USA). For those polymers in spherical pellet form, around 30 mg of pellets (4–6 unities) were put together in a porous metallic bag during the HT process, obtaining a single pellet as a product. In order to generate polymer mixtures (pellet–pellet, powder–powder, or powder–liquid), equal amounts of each polymer were used to total amount of combined polymers: PLGA-PEDOT PSS (30 mg); PVA-PBS (230 mg); PCL-PBS: 230 mg and Chitosan-PBS (180 mg).

The CO_2_ (with a minimum purity of 99.8%) for the foaming experiments and the N_2_ for the hydrothermal treatment were acquired from Linde (Barcelona, Spain).

### 2.2. Hydrothermal Pretreatment (HT)

**Figure 1:**
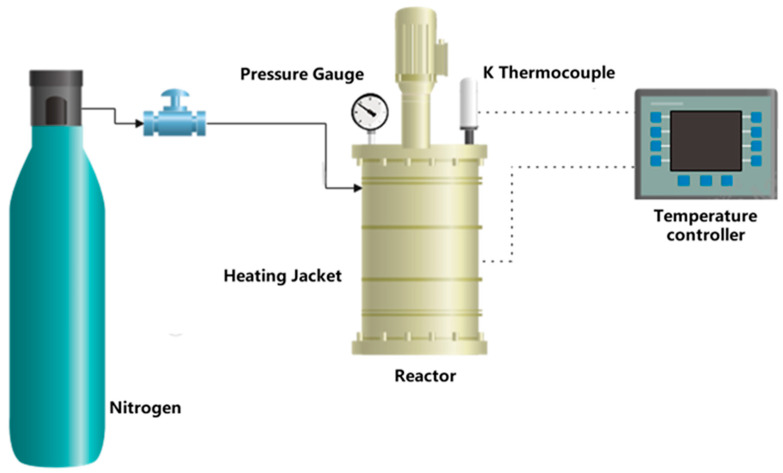
Hydrothermal treatment batch reactor layout.

A Parr 4570 1 L high-pressure reactor (Parr Instrument Company, Moline, Illinois, USA), in batch operation, was used for the hydrothermal tests (Figure 1). This reactor consists of a heating system with a 2300 W cylindrical electric furnace and a temperature probe connected to a regulator. The 316L stainless steel reactor body and head were fitted with flanges for a tight seal, with a pressure gauge (up to 276 bar) and with inlet and outlet valves for gas control.

The reactor was first purged with N_2_, then pressurized and heated to the target conditions. After treatment, the system was cooled to 25 °C and carefully depressurized before retrieving the samples and air-drying them.

### 2.3. Foaming Process Using Supercritical CO_2_ (F)

**Figure 2:**
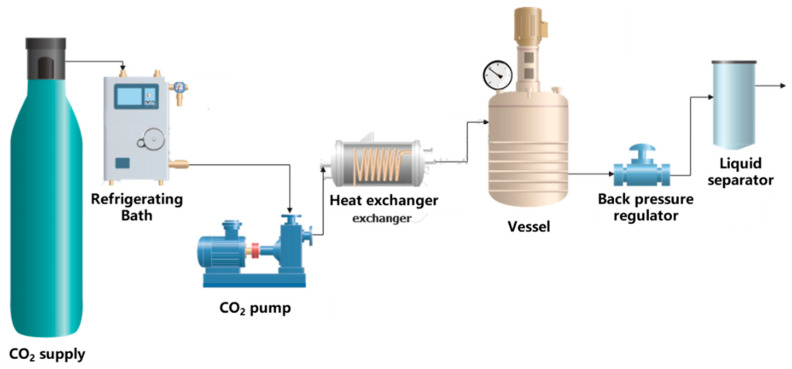
Foaming equipment layout.

The foaming process was carried out in a Thar Technologies RESS250 high-pressure equipment (Thar Technologies Inc., Pittsburgh, Pennsylvania, USA), consisting of a CO_2_ bottle, a condenser to cool the CO_2_, a high-pressure pump to provide the required pressure, a heat exchanger to adjust the temperature, and a stainless-steel vessel where the foaming took place (Figure 2). The equipment was also equipped with a BPR valve to control the depressurization rate.

The foaming step was performed following the methodology previously described in detail by Montes et al. [37]. The tablets were placed in an aluminum foil holder and inserted into a high-pressure chamber. The system was then adjusted to the desired pressure and temperature, and CO_2_ was pumped in until supercritical conditions were reached. After target time, the system was depressurized at a high rate (around 150 bar/min) by means of the BPR.

### 2.4. Physical and Morphological Analysis

An initial screening of the different polymers was performed in order to determine which were the most suitable for the production of scaffolds using the hydrothermal pretreatment process followed by their foaming using supercritical CO_2_. The processing temperature for the HT process was selected to slightly excess the melting temperature of the polymers, and the pressure required to maintain the water in liquid state at that temperature. The processing conditions for the foaming process were chosen based on a previous study [37]. Table 2 and Table 3 show the experiments that were conducted for the initial screening for individual and combined polymers, respectively.

### 2.5. Polymer Screening

#### 2.5.1. Visual Analysis and Expansion Degree

For the first stage of the screening, the external visual aspect of the products was evaluated because it may show surface defects, such as voids, craters, deterioration, or fractionation. Additionally, visual external and internal homogeneity (i.e., the uniformity of the foam’s structure throughout the product) is important for foamed products, as it may be a direct indicator of quality, consistency, and predictable performance. A lack of homogeneity, especially in the internal structure (which is also measured in detail using SEM in Section 2.5.2), means that the product will not perform equally in the different regions, leading to possible failures in critical applications.

**Figure 3:**
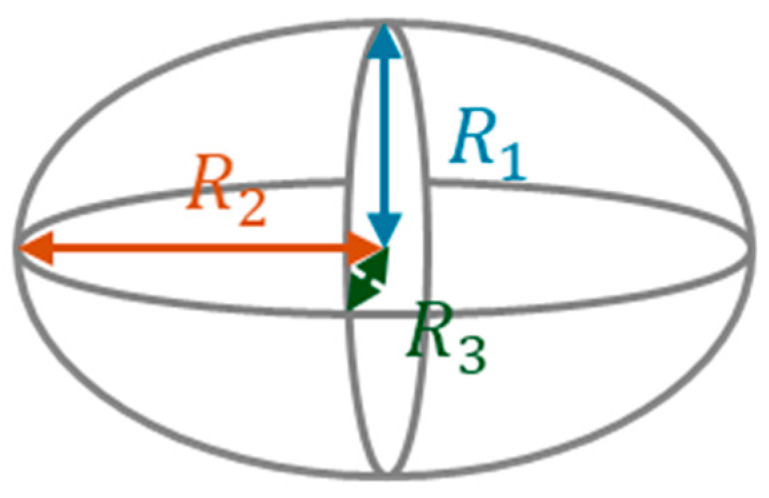
Particle radii used for volume calculation for initial samples and products.

The expansion degree (also known as the expansion factor or foaming ratio) is an important parameter in any foaming process because it is related to physical properties (density, porosity), performance characteristics (mechanical properties), and the suitability of the resulting foam product for its intended application. The expansion degree was calculated as the ratio of the final volume of the foam to the initial volume of the polymer material used to create it. The polymer samples that were obtained after the hydrothermal pretreatment and foaming process presented an irregular, slightly oval shape. Therefore, their volume was calculated as that of an ellipsoid, instead of a sphere, to achieve a more accurate measurement. A caliper was used to measure three dimensions of each sample, and the formula for the volume of an ellipsoid was applied:

(1) $$Ellipsoid\;volume=\frac{4}{3}\pi R_{1}R_{2}R_{3}$$

where *R*_1_, *R*_2_, and *R*_3_ correspond to the radii of the measured space dimensions, as shown in Figure 3.

The volume was registered for each stage of the process and the expansion degree, which reflects the volume increase under the different conditions, was calculated using Equation (2):

(2) $$Expansion\;degree=\frac{Final\;volume}{Initial\;volume}$$

Multiple particles were measured for each test, yielding a standard deviation of less than 5% for compacted samples, while, for spherical samples provided in pellet form, the standard variation did not exceed 10%.

#### 2.5.2. Scanning Electron Microscopy

Scanning Electron Microscopy (SEM) was used to examine in detail the external and internal morphology and homogeneity of all samples and products. For internal evaluation, the solid product was mechanically cut and a cross section of each sample was selected. A Nova NanoSEM 450TM microscope from the Central Scientific Research and Technology Services (SC-ICYT) at the University of Cadiz was used.

The polymer samples treated with supercritical CO_2_ and hydrothermal treatments were previously coated with a 10 nm layer of gold in order to improve their conductivity.

## 3. Results

Subcritical water, often referred to as hot compressed water, is water maintained at elevated temperatures (100–374 °C) and high pressures (typically 1–22 MPa) to keep it in the liquid state. In this state, water exhibits unique properties such as reduced dielectric constant, increased ion product, and enhanced diffusivity, which produce several physical (glass transition, melting, swelling, erosion, etc.) and chemical (hydrolysis and other reactions) effects in the surface, structure, and composition of biodegradable polymers. The different effects produced in different polymers depend on the operation conditions used (mainly temperature and pressure) and nature of the polymer (glass transition temperature (T_g_), melting temperature (T_m_), crystallinity, hydrophilic behavior, hydrolysable linkages, etc.). Temperatures around the melting point of the polymer can produce desirable changes in its surface and structure, such as crystallinity reduction and pore formation, which subsequent enhance the foaming process with CO_2_. However, high temperatures may lead to an excessive increase in the kinetic energy of water molecules and polymer chains, speeding up the rate of chemical or thermal degradation.

This study involves the screening of a number of polymers where a hydrothermal pretreatment followed by a supercritical CO_2_ (scCO_2_) foaming process was evaluated. The effects of this sequence of treatments on the stability, homogeneity, morphology, and porosity of the resulting scaffolds were analyzed. Polymers that failed the screening test (severe degradation, lack of homogeneity, pitting, etc.) are directly discarded for further steps. The experiments were carried out with both individual polymers and polymer combinations, which allowed us to identify those polymers that are suitable for an HT step—polymers presenting reasonable potential for property improvement—which were then selected for a more detailed study in part 2.

**Figure 4:**
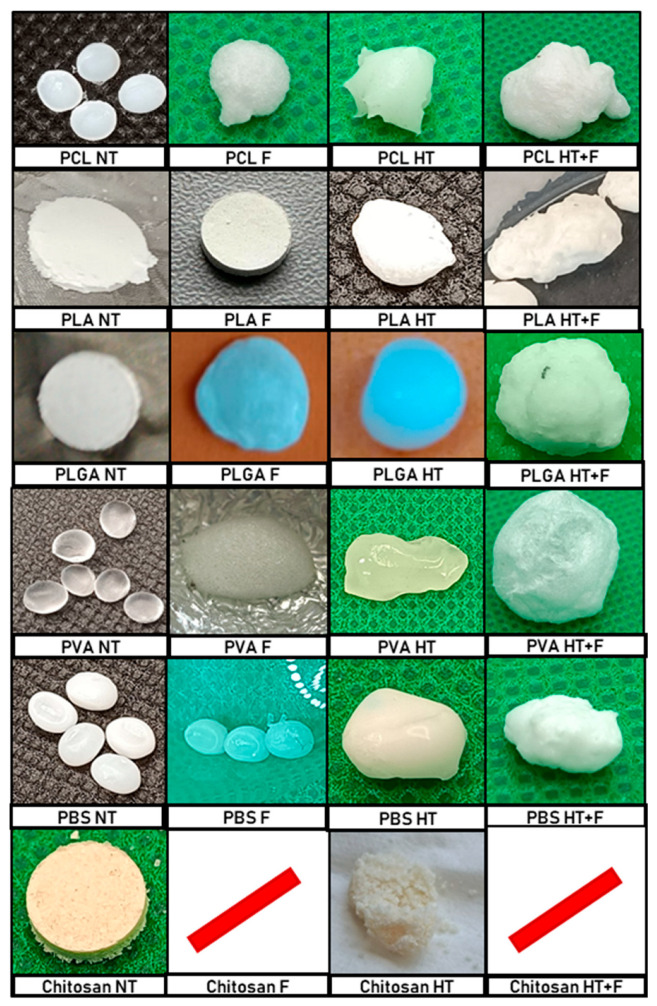
Morphology of the individual polymers at each stage of the process (NT: Non-treated polymer; F: Foaming without hydrothermal pretreatment; HT: Only hydrothermal pretreatment; HT + F: Foaming after hydrothermal pretreatment). (/) denotes a lack of product due to structural stability loss and severe material loss.

Figure 4 contains all the images corresponding to the different stages of the treatment of the samples analyzed based on the operating conditions listed in Table 2. Figure 4 shows the initial samples and the corresponding products depending on the treatment route applied. The first column shows a picture of the samples (non-treated, NT). This column displays two distinct sample preparations: (1) Several raw commercial polymer pellets that will be treated together, and (2) pressed pellets prepared at the laboratory from commercial powder. The second column includes the foamed product (F) obtained from the NT polymer. The third column includes the product obtained after the hydrothermal treatment (HT), and the fourth column shows the products obtained by foaming the hydrothermally pretreated sample (HT + F).

The images in Figure 4 show that, for the PCL, PLGA, PVA, and PBS samples, the HT treatment was successful, and a single apparently highly homogeneous conglomerate was obtained where the polymer samples had fused together. Furthermore, when these conglomerates were subjected to a subsequent foaming process, there was no apparent deterioration, fractionation, or sample loss, while the volume of the conglomerate had considerably increased. The results are shown in Section 3.1. Furthermore, it is necessary to verify that an adequate internal porous structure had been created, which can be evaluated with the help of SEM images. These results are shown in Section 3.2. On the other hand, it should be noted that, in the case of PLA, the HT + F product appeared broken into three pieces, with the biggest one used for the SEM analysis, although the polymer will be discarded for further tests. In the case of chitosan, the sample completely lost its structural stability during all of the steps, which prevented the entire process from being carried out.

**Figure 5:**
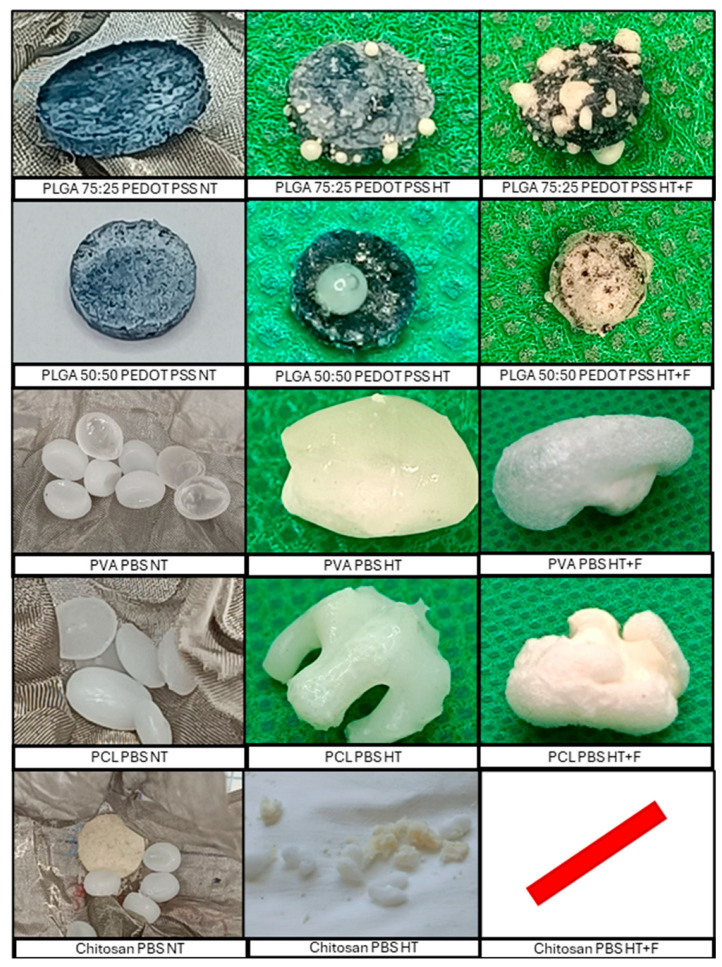
Morphology of the combined polymers at each process stage (NT: Non-treated sample; HT: With thermal pretreatment; HT + F: Foaming after thermal pretreatment). The (/) symbol means that the process is not applicable due to the lack of a proper combination of both polymers.

In turn, Figure 5 shows the initial materials and the products obtained with the process route applied. The first column shows the picture of the samples (Non-treated, NT): (1) raw commercial pellets of two different polymers that are introduced together in the HT reactor; (2) pressed pellets prepared at the laboratory, mixing the polymer in powder with the liquid polymer; (3) raw commercial pellets of one polymer and prepared pellet from the second polymer (powder) that are introduced together in the HT reactor. The second column includes the product obtained after the hydrothermal treatment (HT). The third column shows the products obtained by foaming the hydrothermally pretreated sample (HT + F). In the case of combined polymers, no foaming tests were carried out for the NT polymer, since it is necessary for the HT treatment to obtain a combined polymer via a fusion of both individual polymers.

In most cases, HT treatment successfully joined together both polymers, producing a single solid including both components. However, a very high degree of heterogeneity was observed in the outer surface of the solid product for combined polymers with PLGA. In this case, the temperature used (always below 200 °C) is not sufficient to melt that polymer (T_m_). In the case of PCL and PVA, the polymers melt together, but some areas have a different color or different brightness that corresponds to a clear predominance of a specific polymer. Furthermore, in the case of chitosan + PBS, the hydrothermal treatment did not manage to combine the two polymers at all, so the test was excluded from the complete process (HT + F). In most cases, for combined polymers, the evaluation of the external visual aspect reveals surface defects as voids, craters, deterioration and lack of homogeneity, but the evaluation of the expansion degree and SEM analysis are still required to select or reject the products.

### 3.1. Determining the Expansion Degree of the Resulting Structures

This parameter allows us to quantify the swelling or degree of expansion induced by the hydrothermal pretreatment and/or the foaming process with supercritical CO_2_.

#### 3.1.1. Expansion Degree of Single Polymers

**Figure 6:**
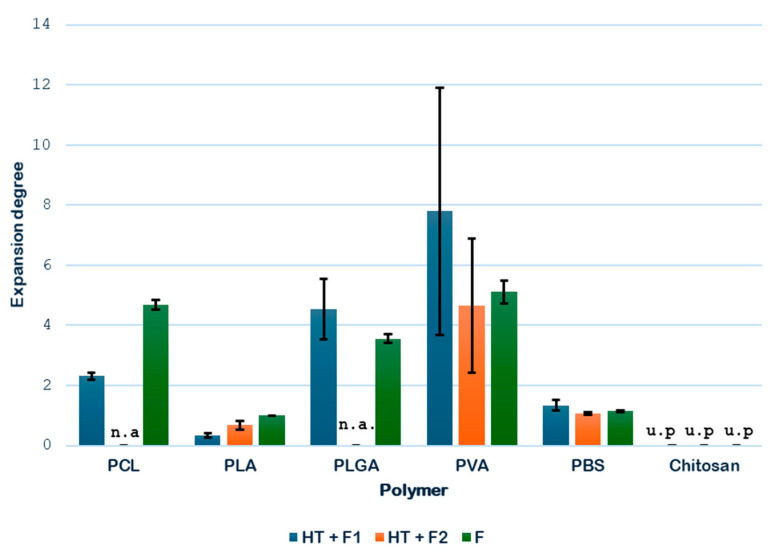
Single polymer expansion degree (n.a.—not available; u.p.—unsuitable product).

Figure 6 compares the expansion factors determined for each single polymer when subjected to the scCO_2_ foaming process either with or without the hydrothermal pretreatment.

Based on the results obtained, it can be stated that, for PCL, the route NT + F gives a better expansion degree (4.67) than that obtained in the novel route HT + F (2.31), where the HT was carried for the first time at a temperature of 150 °C. It should be noted that the foaming without thermal pretreatment was applied to a single-pellet sample, while the process that included the thermal pretreatment was applied to a four-pellet sample, which formed a conglomerate after the thermal treatment. It is a fact that, due to its high diffusivity and low viscosity, supercritical CO_2_ penetrates more easily into materials with a smaller mass, which offer less resistance to gas diffusion and favor a greater porosity and specific area. For samples of equal mass to be compared, it would be necessary to produce in advance a four-pellet PCL agglomerate using a solvent, since the foaming with CO_2_ does not melt the material to agglomerate, and the use of an organic solvent is avoided in this work. The decision to use four-pellet samples was justified by the need to have a sufficient amount of material for the biocompatibility and mechanical characterization (specifically, the resistance to compression, which is important for validating the scaffolds for tissue engineering) tests to be carried out in later phases of this study. In any case, the novel route is considered suitable for this PCL even with an HT temperature of 150 °C. The results obtained at different temperatures are to be discussed in Part 2.

Regarding the expansion degree registered for the PLA samples, the best results for HT + F were obtained when the foaming temperature was increased from 40 °C (F1) to 70 °C (F2). This makes sense, since it slightly exceeds the glass transition temperature (T_g_) of PLA (~60 °C), thus promoting a greater expansion of the polymer, which reached a factor of 0.68. Although the route NT + F gives a higher expansion rate, SEM analysis will be evaluated in Section 3.2 to conclude the initial suitability or not of the novel route (HT + F).

In the case of PLGA, previous studies using only supercritical CO_2_ obtained good foaming results with PLGA 75:25, showing an expansion degree around 3.55 [34]. The present work started with the application of the novel route HT +F with that polymer, improving the previous results with a significant expansion of 4.54. PLGA presents a very high T_g_ (60 °C), a temperature only exceeded in the HT treatment, producing a transition from a hard and glassy to a soft and rubbery state of the polymer. Water molecules penetrate the polymer surface and diffuse, so the polymer swells but does not dissolve unless degradation (mainly by hydrolysis) progresses far enough. HT-treated samples present a more spherical shape that favors contact with CO_2_ and improves its diffusion.

On the other hand, in the case of PVA, the same trend as for PCL was observed: the sample that had not been subjected to the hydrothermal pretreatment presented a greater volume growth. Even though PVA is highly hydrophilic, its resistance to degradation in hot compressed water comes from other structural and chemical factors. It does not contain ester bonds (which are easily hydrolyzed), but it has a chemically stable carbon–carbon backbone (which is not hydrolysable) and it forms extensive hydrogen bonding that makes it resistant to disruption even in hot compressed water.

Finally, the results for PBS were also favorable. Some expansion was observed after applying the hydrothermal treatment followed by foaming, with a volume increase of up to 1.34, especially at lower temperatures (45 °C), which are closer to its T_g_. In comparison, the sample without hydrothermal pretreatment exhibited a poorer performance, with a factor of only 1.15.

At this stage of the screening process, only chitosan is discarded for the novel route due to the complete degradation during the HT treatment, due to a combination of hydrolytic and thermal degradation mechanisms. Chitosan is a natural polysaccharide, primarily composed of β-(1→4)-linked D-glucosamine units (and some N-acetyl-D-glucosamine). It has glycosidic bonds between sugar units. These bonds are susceptible to hydrolysis, especially under acidic or high-temperature conditions. Even though the HT experiment was carried out at a low temperature (110 °C, which is slightly above its Tm), the degradation proceeds rapidly and extensively, leaving no intact polymers.

#### 3.1.2. Expansion Degree of the Combined Polymers

**Figure 7:**
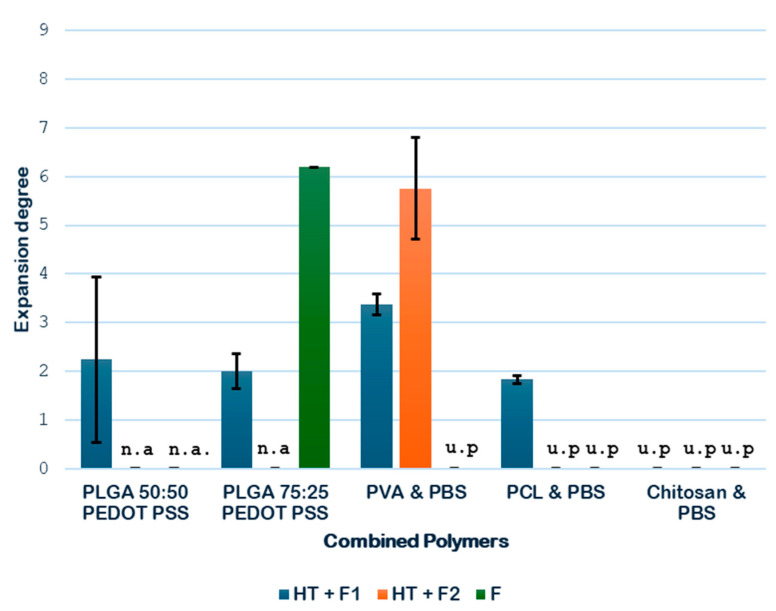
Combined polymer expansion degree (n.a.—not available; u.p.—unsuitable product).

Next, the expansion degrees of the combined polymers considered in the study were determined, and the results are shown in Figure 7. In the case of combined polymers, the pretreatment with HT is also used to melt and join both polymers together, thanks to the high temperature achieved. For this reason, the direct foaming of the raw mixture of the polymer (NT + F) could not be tested.

As shown in Figure 7, the hydrothermal treatment (HT) contributed to an increment in the volumetric expansion of the samples analyzed. In the case of PLGA-PEDOT: PSS, two combinations are studied to analyze it, in addition to the previously studied 75:25; good results can be obtained with 50:50. A greater expansion was obtained with the PLGA 50:50 formulation in comparison with the PLGA 75:25 formulation, with expansion degrees of 2.25 and 2.01, respectively. These results suggest that a more balanced ratio between monomers may favor the formation of more porous structures when the combined HT and foaming treatments are applied.

With regard to the PVA PBS combination, a significant enhancement of the expansion was observed when the pressure was increased during the foaming process, as it went up from 3.37 to 5.75. This enhancement could be explained by a shift in the glass transition temperature (T_g_) of this combination, the estimated value of which lies between the T_g_ of each of its polymers (i.e., between −25 °C and 80 °C), which is probably closer to the operating temperature (45 °C), thus promoting a phase of greater permeability to CO_2_ of the combined material. Finally, regarding the PCL PBS combination, the results confirmed a remarkable expansion of 1.83 when the hydrothermal treatment followed by the foaming process was applied.

At this stage of the screening process, the combined polymers PLGA 75:25-PEDOT: PSS and chitosan-PBS are discarded for the novel route due to the complete heterogeneity of the former and to the complete degradation during the HT treatment.

Even though HT has fostered the selected polymers’ swelling, it is important to check the porosity of the structure generated. The purpose of the following part of the study is to evaluate the effect of the initial hydrothermal treatment on the morphology of the resulting scaffold. Certain characteristics, such as the formation of macropores, are essential to obtain an optimal scaffold structure. Each stage of the process was analyzed and, in the case of single polymers, a control sample that was not subjected to hydrothermal treatment was also studied.

### 3.2. Scanning Electron Microscopy (SEM) for Single Polymers

**Figure 8:**
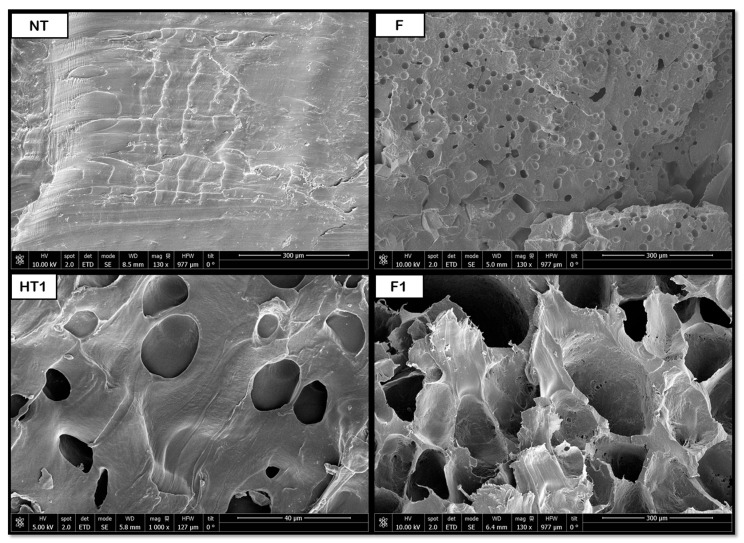
Internal porosity PCL (NT, non-treated; F, foaming without pretreatment; HT1, hydrothermal treatment test 1; F1, foaming of the product obtained from HT1).

**Figure 9:**
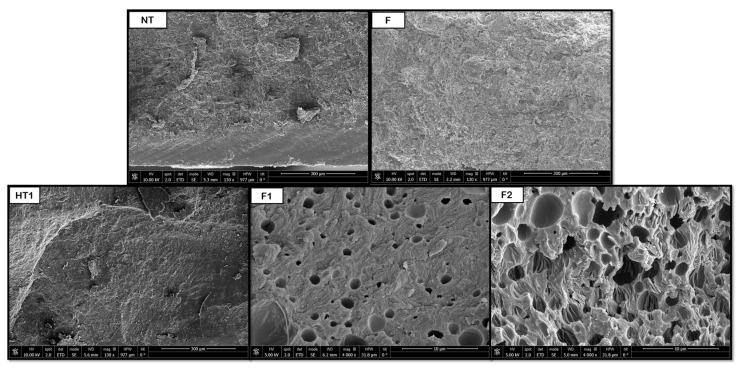
Internal porosity of PLA (NT, non-treated; F, foaming without pretreatment; HT, hydrothermal treatment; F1, foaming test 1; F2, foaming test 2).

**Figure 10:**
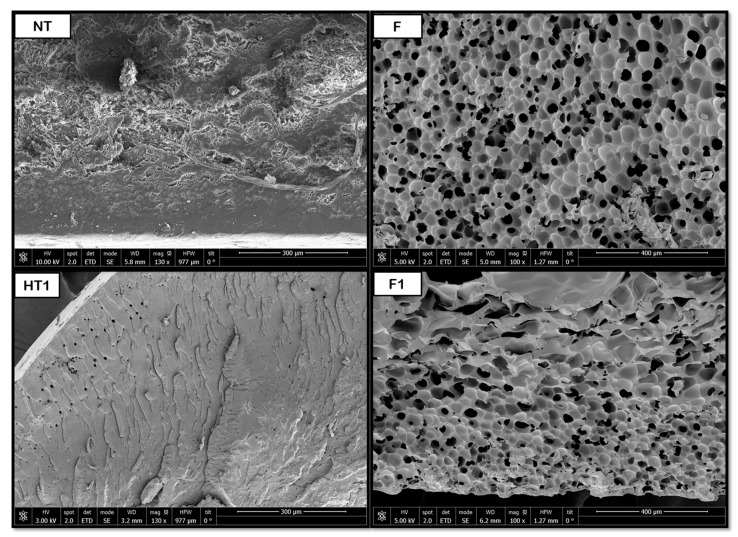
Internal porosity of the PLGA (NT, non-treated; F, foaming without pretreatment; HT1, hydrothermal treatment test 1; F1, foaming test 1).

**Figure 11:**
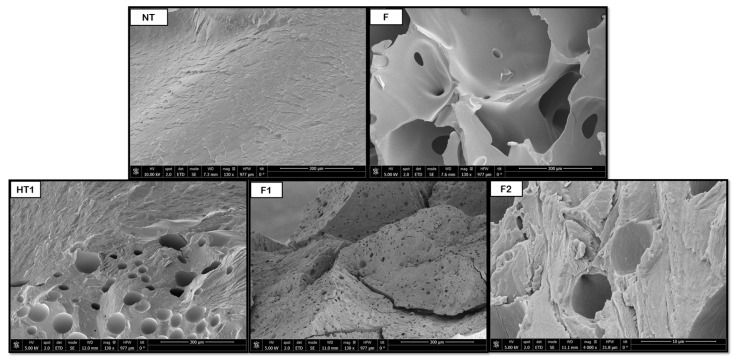
Internal porosity of the PVA (NT, non-treated; F, foaming without pretreatment; HT1, hydrothermal treatment test 1; F1, foaming test 1; F2, foaming test 2).

**Figure 12:**
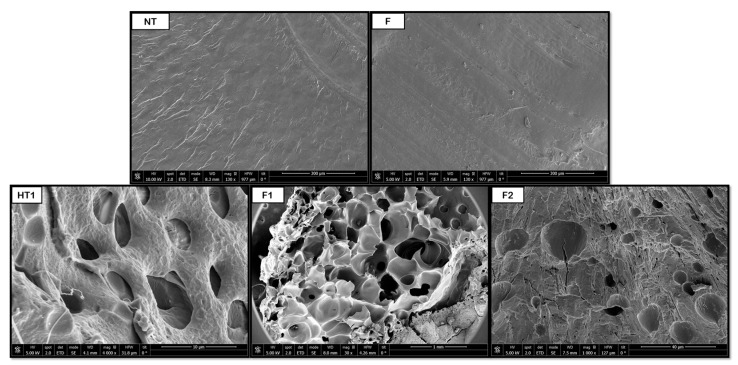
Internal porosity of the PBS (NT, non-treated; F, foaming without pretreatment; HT, hydrothermal treatment; F1, foaming test 1; F2, foaming test 2).

Figure 8, Figure 9, Figure 10, Figure 11 and Figure 12 show some of the images obtained using Scanning Electron Microscopy (SEM), which allowed us to observe in detail the morphology of the surface and the distribution of the pores in the raw and treated samples for individual polymers.

**Figure 13:**
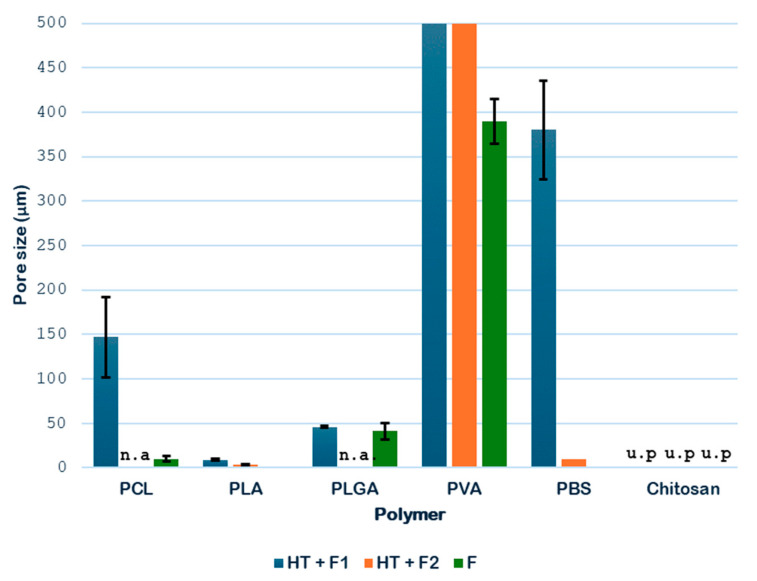
Graph showing the pore diameter of the single polymers (n.a.—not available; u.p.—unsuitable product).

Figure 13 shows the pore sizes of the different single polymer samples obtained.

The data shown in Figure 13 indicate that the hydrothermal treatment applied to PCL promotes the formation of porous cavities, especially inside the samples. Subsequently, when applying the supercritical CO_2_ foaming technique, a significant increase in the average pore size is observed, reaching approximately 147 μm. The novel route generates a promising product, showing a homogeneous pore distribution, as shown in Figure 8, where a significant increase in size and even distribution can be observed. All in all, the data show that the application of pre-HT improves the porous morphology of the scaffolds compared to that of the samples that were just foamed. These results proved to be consistent when compared against those obtained from previous experiments by the same research team, which involved hydrothermal treatment at 100 °C, 17 bar, and 10 min, followed by foaming at 40 °C, 300 bar, and 1 h with rapid depressurization, where pore sizes of 170 μm were obtained [35]. This enhancement in pore formation can be attributed to the hydrothermal pre-treatment, facilitating partial polymer chain relaxation and crystallinity reduction, which improves CO_2_ diffusion during foaming. The presence of water at elevated temperature and pressure likely promotes localized hydrolysis and surface plasticization, creating nucleation sites that favor gas penetration and bubble growth. Consequently, the observed homogeneous pore distribution may result from the balance between diffusion kinetics and polymer viscosity during the supercritical foaming stage.

As for the PLA products, porous structures were observed at a temperature of 160 °C during hydrothermal treatment. When the foaming process was applied, the product broke into three pieces, so the novel route was discarded for further experiments. The results may be explained by the brittleness of PLA, which may suffer hydrolysis of ester bonds during HT, combined with the internal stresses created by the rapid depressurization during the foaming process. Nevertheless, the main piece was analyzed and the pore distribution improved, even though no significant variations in pore size were detected, remaining at around 7 μm. This behavior can be seen in Figure 9, where a substantial improvement in pore distribution can be observed when comparing HT to F1 and F2 results. This can be compared with the results obtained by Peng et al. [38] from a PLA foaming test using CO_2_ extrusion at 27 °C, 60 bar, and 24 h with rapid depressurization, pore diameters between 4 and 14 μm were obtained. The limited change in pore size, despite improved distribution, suggests that the hydrothermal treatment primarily influenced nucleation density rather than gas expansion dynamics in PLA.

In the case of PLGA samples, from Figure 10, it was determined that the hydrothermal treatment modified pore formation, although it is not completely homogeneous. However, these pores increase in size after the foaming process, with pores in the range of 40–50 μm, with a more homogeneous distribution throughout the product. Compared to the samples without pre-HT (41 μm), the pore sizes were rather similar, so clear benefits of the novel route could not be justified until the tomography analysis are evaluated. In terms of comparison with existing literature, the results obtained by Álvarez et al. [39] in a PLGA CO_2_ foaming test using ethyl lactate polymer solutions at 40 °C, 200 bar, and 24 h with rapid depressurization were taken, and the diameter of the pores obtained was between 35 and 158 μm. PLGA’s copolymeric composition and degradation behavior under hydrothermal conditions could explain the moderate increase in pore size. The hydrothermal treatment may selectively affect the glycolic segments, reducing crystallinity and enhancing the gas solubility during foaming. However, the modest effect observed indicates that phase separation and CO_2_ diffusion were limited by polymer viscosity or residual crystallites. Compared to solvent-assisted systems, the absence of plasticizing agents may restrict pore coalescence, explaining why the pores remained within a relatively narrow size range.

With regard to PVA, the hydrothermal treatment promoted the formation of initial pores, but non-porous regions, and therefore heterogeneity, can be clearly observed in the SEM image. The subsequent foaming appeared to minimally reduce the average size of the pores, although it significantly improved the number of pores and their distribution. This apparent reduction in pore size arose because the polymer expands its volume in such a way that the internal cavities are transformed into larger pores, as shown in Figure 11. When comparing the HT samples against those without pretreatment, internal pores larger than 500 μm were obtained in the former, while the latter presented around 390 μm pores. These results were compared against those obtained by Yin et al. [40] in a PVA foaming test with CO_2_ together with a plasticizing agent, ethylene glycol, at 225 °C, 200 bar, and 2.5 h with rapid depressurization, where the diameter of the pores obtained was between 9 and 13 μm. The contrasting behavior of PVA can be linked to its hydrophilic nature and high degree of hydrogen bonding. Hydrothermal exposure likely disrupts crystalline domains and enhances chain mobility, facilitating expansion during CO_2_ saturation. The apparent decrease in pore size following foaming might therefore reflect the collapse of weaker pore walls or redistribution of gas within the polymer matrix. Moreover, the absence of an external plasticizer may have limited bubble stabilization, leading to coalescence and reorganization of pores.

In the case of PBS, the hydrothermal treatment was essential to inducing the formation of pores with a good distribution. For this polymer, the foaming process of non-treated samples only showed a very reduced pore formation. As shown in Figure 12, the pores only appeared clearly in F1 and F2 after HT. In addition, a greater pore expansion was detected at lower foaming temperatures (45 °C), with sizes up to 380 μm. If these results are compared against those obtained by Ju et al. [41] in a PBS foaming test with CO_2_ together with a mixture of solvents using an electrospinning technique (chloroform and methanol) at 110 °C, 220 bar, and 2 h with rapid depressurization, the diameter of the pores obtained was between 20 and 140 μm. The strong temperature dependence of pore expansion in PBS suggests that lower foaming temperatures favor controlled CO_2_ desorption, allowing gradual bubble growth and stabilization. Hydrothermal treatment likely enhances this effect by reducing the polymer’s crystallinity and increasing free volume, thus improving gas solubility and diffusion. The markedly reduced presence of pores in non-treated samples supports the hypothesis that the hydrothermal pre-treatment acts as a structural “activator,” generating amorphous regions where gas can nucleate. The larger pore sizes compared with solvent-assisted methods indicate that the novel route proposed in this work is very promising for further studies, effectively producing highly porous PBS structures without residual solvents, which is advantageous for biomedical use.

### 3.3. SEM for Combined Polymers

**Figure 14:**
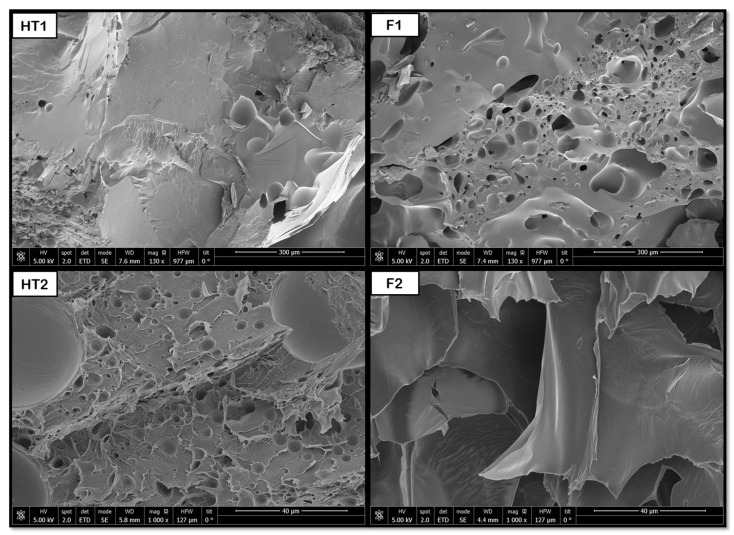
Internal porosity of the PLGA and PEDOT: PSS composite (HT1 and F1 for PLGA 50:50); (HT2, and F2 for PLGA 75:25).

**Figure 15:**
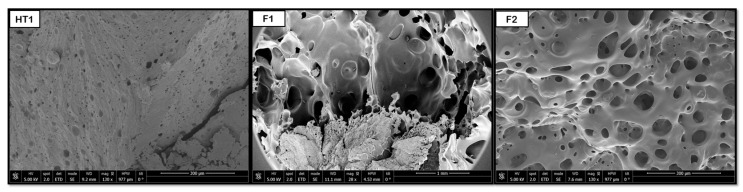
Internal porosity of the PVA and PBS composite (HT1: Hydrothermal Treatment test 1, F1: Foaming test 1, F2: Foaming test 2).

**Figure 16:**
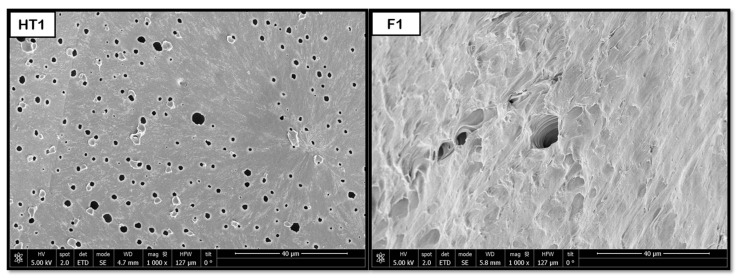
Internal porosity of the PCL and PBS combination (HT1: Hydrothermal Treatment test 1, F1: Foaming test 1).

Below in Figure 14, Figure 15 and Figure 16 are the main SEM images of raw and treated samples (see operating conditions in Table 3) for combined polymers.

**Figure 17:**
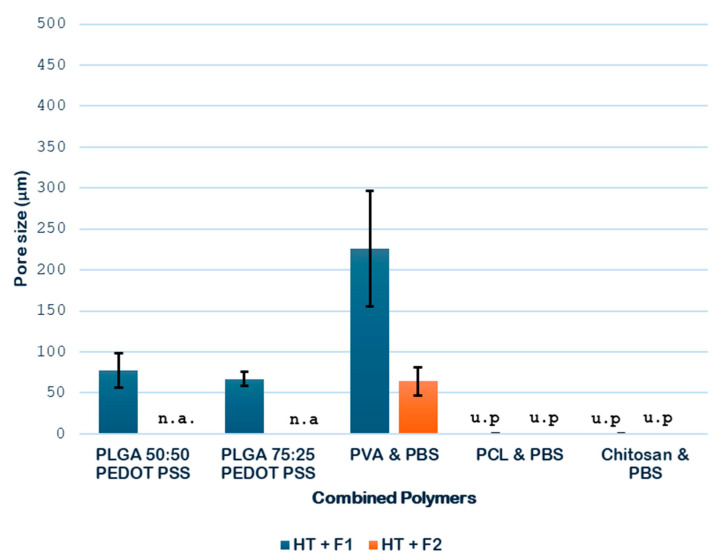
Graph of the pore diameter of the combined polymers obtained (n.a., not available; u.p., unsuitable product).

The data obtained in the case of combined polymers are analyzed in Figure 17.

The hydrothermal treatment has been shown to promote the formation of pores in PLGA and PEDOT polymer samples with both proportions (50:50 and 75:25). Figure 14 shows that some pores are formed after the HT process, resulting in diameters around 2 μm for 50:50 and 3 μm for 75:25. However, the novel route produces a considerable increase in pore sizes, up to 77 (50:50) and 67 μm (75:25), and an improvement in their distribution. The hydrothermal environment subjects the polymers to hydrolysis. This hydrolysis reduces the polymer’s molecular weight, which in turn causes a decrease in the melt viscosity during the subsequent foaming process. A lower melt viscosity facilitates the diffusion-driven growth of the scCO_2_ to much larger diameters with the novel route. Due to the heterogeneity of the products obtained, only a slight increase can be established, since it will vary depending on the area we select for analysis. Therefore, although the initial pores generated by the HT act as precursor heterogeneous nucleation sites, these pores ∼70 μm are smaller than those reported by Montes et al. [37] for a foaming test with CO_2_ on PLGA 75:25-PEDOT: PSS at 45 °C, 210 bar, and 30 min with rapid depressurization, where the diameter of the pores obtained was 128 μm.

In the case of combined PVA and PBS samples, the hydrothermal treatment has also been proven to induce pore formation. After foaming, a notable increase in pore size and a more even distribution could be observed, as shown in Figure 15, where the transition between just applying HT and the combination HT + F is clearly visible. The significant pore size increase in the PVA/PBS products is consistent with the accelerated growth mechanism outlined above. However, the observation that the largest average internal pore size (∼227 μm) was obtained at the lower foaming pressure (200 bar) goes against conventional foaming theory and highlights the critical role of blend heterogeneity.

On the other hand, the hydrothermal treatment promoted pore formation in the combined PCL and PBS samples, although the pores were small in size. With the later application of the foaming technique, a smaller number of pores is obtained, but they were larger, as shown in Figure 16. This apparent loss of pores could be attributed to the heterogeneity of the sample, as well as the cutting area selected for the analysis.

Regarding this screening work, the production of highly homogeneous products has been considered a key factor in selecting a polymer as promising; therefore, with the exception of PCL-PBS, combined polymers will be discarded for further experiments. However, that mixed-porosity product could have several biomedical applications due to its heterogeneous structure, so these products may be interesting for other studies. For example, the combination of porous and non-porous regions could be strategically used to meet different biological requirements simultaneously for drug delivery systems (where non-porous regions can act as a structural barrier or a slow-release matrix), tissue engineering scaffolds (non-porous regions can provide extra mechanical strength and structural support to the scaffold), etc.

Considering all of the information presented above, Table 4 contains the polymers studied and their optimal processing conditions, which have been classified as promising (symbol “√√“ denotes a product of great interest for a further study), suitable (symbol “√“ denotes an applicable process), or non-suitable (lacking interest for further studies). The table also includes their potential biomedical applications. The table shows that materials yielding high porosity (~80–90%) with large, interconnected pores are best suited for bone or cartilage regeneration [42] (due to vascularization and large cell migration needs) as happens with PVA and PBS, which exhibited highly porous structures with high expansion degrees (7.80 and 1.35, respectively) and large pore sizes (>500 µm and 380 µm). In contrast, PLA generated a much denser structure with a low expansion degree (0.33) and a small pore size (7 µm), making it better suited for connective tissue cells in the growth phase or controlled drug delivery systems [43,44].

## 4. Conclusions

This study tested the suitability of a novel route to producing improved polymers based on hydrothermal pretreatment followed by supercritical CO_2_ foaming for various individual polymers (PCL, PLA, LGA, PVA, PBS, and chitosan) and combined polymers (PCL-PBS, chitosan-PBS, PVA-PBS, and PLGA-PEDOT: PSS).

It has been proven that the novel route proposed, which avoids the use of organic solvents, was not suitable for generating suitable combined polymers from the individual polymers under the conditions studied. The resulting products present several areas with good porous structures, but with very low homogeneity and low repeatability, as they varied depending on the region where one of the polymers predominates. This is particularly true when combining PLGA with PEDOT: PSS, PVA with PBS, PCL with PBS, and chitosan with PBS.

More satisfactory results have been obtained with all individual polymers, which, except for chitosan, presented a porous structure after the process was completed. In addition, PCL, PLGA, PVA, and PBS polymers may be the most appropriate candidates for applications in biomedicine, as they present the most promising products regarding homogeneity, expansion degree, and a very wide pore distribution. For these reasons, in the second part of this study, these polymers are further investigated, testing more operating conditions for HT and F or conducting further analyses of the products, such as mechanical testing and X-ray tomography, to characterize the mechanical and structural properties of the individual polymers in greater depth.

## Figures and Tables

**Table 1:** Biomedical applications according to polymer properties, adapted from Kosowska et al. [8].

Property	Parameter	Range of Variation	Application	References
Porosity	Porosity	80%	Biomedical engineering	[12,13,14,15,16]
Morphological	Pore size	5 µm	Newly formed tissue	[17]
		5–15 µm	Proliferating connective tissue cells	
		20 µm	Hepatocytes	
		20–125 µm	Adult person skin	
		100–350 µm	Bone tissue	
		>500 µm	Fibrovascular tissue	
		Micropores	Tissue engineering	[18]
		100 µm	Bone tissue	[12]
		450 µm	Bone tissue	[19]
		<100 µm	Connective tissue	
		Micropores (<50 µm) and macropores (150–300 µm)	Fibrocartilage	
		<100 µm	Bone tissue	[20]
		100–500 µm	Blood vessels	
		200–350 µm	Bone tissue	[14]
		100–350 µm	Bone tissue	[17]
Mechanical	Young’s modulus	7–30 GPa	Bone structure	[13,21,22]
		0.02–0.8 GPa	Spongy bone	
	Mechanical resistance	100–230 MPa	Bone structure	
		2–12 MPa	Spongy bone	
Biological	Biocompatibility	Optimal non-toxic tolerance without genetic mutations in the surrounding cells and without any inflammation	Biomedical engineering	[13,23,24,25,26,27]
	Biodegradability	The degradation rate of the implanted material has been adjusted to the regeneration rate of the damaged tissue	Biomedical engineering	[13,23,27]

**Table 2:** Operation conditions for single polymer screening.

Polymer	T_g_ (°C)	T_m_ (°C)	Hydrothermal Pretreatment (HT)	Foaming with CO_2_ (F)
PCL (pellets)	−60	60	150 °C 25 bar 30 min	40 °C 300 bar 1 h
PLA (powder)	60	150	160 °C 25 bar 10 min	F1: 40 °C 300 bar 1 h
				F2: 70 °C 300 bar 1 h
PLGA (75:25) (powder)	45–60	225–262	100 °C 25 bar 10 min	45 °C 210 bar 30 min
PVA (pellets)	75–85	190	190 °C 25 bar 10 min	F1: 50 °C 200 bar 1 h
				F2: 50 °C 300 bar 1 h
PBS (pellets)	(−40)–10	90–120	120 °C 25 bar 10 min	F1: 45 °C 250 bar 1 h
				F2: 70 °C 250 bar 1 h
Chitosan (powder)	50	102.5	110 °C 25 bar 10 min	Unsuitable

**Table 3:** Operation conditions for combined polymer screening.

Polymer	T_g_ (°C)	T_m_ (°C)	Hydrothermal Pretreatment (HT)	Foaming with CO_2_ (F)
PLGA 50:50 (powder) PEDOT: PSS (liquid)	PLGA: 40–50 PEDOT: PSS: 70	PLGA: 225–262 PEDOT: PSS: 100	135 °C 25 bar 10 min	45 °C 210 bar 30 min
PLGA 75:25 (powder) PEDOT: PSS (liquid)	PLGA: 45–55 PEDOT: PSS: 70	PLGA: 225–262 PEDOT: PSS: 100		
PVA (pellets) PBS (pellets)	PVA: 75–85 PBS: (−40)–(−10)	PVA: 190 PBS: 90–120	190 °C 20 bar 10 min	F1: 45 °C 200 bar 1 h
				F2: 45 °C 300 bar 1 h
PCL (pellets) PBS (pellets)	PCL: −60 PBS: (−40)–(−10)	PCL: 60 PBS: 90–120	120 °C 25 bar 30 min	40 °C 300 bar 1 h
Chitosan (powder) PBS (pellets)	Chitosan: 50 PBS: (−40)–(−10)	Chitosan: 102.5 PBS: 90–120	110 °C 25 bar 10 min	Unsuitable

**Table 4:** Optimal processing conditions of the polymers studied and their potential applications.

Polymer	Hydrothermal Treatment	Foaming	Homogeneity	Expansion Degree	Pore Size (μm)	Potential Applications	Screening Evaluation
PCL	150 °C 25 bar 10 min	40 °C 300 bar 1 h	Homogeneous	2.31	147	Bone tissue, blood vessels and fibrocartilage tissue	√√
PLA	160 °C 25 bar 10 min	70 °C 300 bar 1 h	Homogeneous	0.33	7	Connective tissue cells in growth phase	√
PLGA 75:25	100 °C 25 bar 10 min	45 °C 210 bar 30 min	Homogeneous	4.54	46	Adult human skin cells, connective tissue cells, fibrocartilage tissue, and bone tissue	√
PVA	190 °C 25 bar 10 min	50 °C 200 bar 1 h	Homogeneous	7.80	>500	Fibrous vascular tissue	√
PBS	150 °C 25 bar 1 min	-	Homogeneous	1.35	380	Connective tissue cells in the growth phase and newly formed tissue	√√
Chitosan	110 °C 25 bar 10 min	-	Homogeneous	-	-	-	Not Suitable
PLGA 50:50 PEDOT: PSS	135 °C 25 bar 10 min	45 °C 210 bar 30 min	Heterogeneous	2.25	77	Growth of adult human skin and connective tissue	Not Suitable
PVA PBS	190 °C 20 bar 10 min	50 °C 200 bar 1 h	Heterogeneous	3.37	227	Fibrocartilage and bone tissue	Not Suitable
PCL PBS	150 °C 25 bar 10 min	40 °C 300 bar 1 h	Heterogeneous	2.30	-	-	Not Suitable
Chitosan PBS	110 °C 25 bar 10 min	-	Heterogeneous	-	-	-	Not Suitable

√ The polymer properly supports the HT process. Product structure using the novel route does not clearly improve the direct foaming with CO_2_. √√ Product structures obtained using the novel route are promising because they clearly improve the direct CO_2_ foaming. Further HT conditions and detailed characterization will be tested in Part 2.

## Data Availability

Data are contained within the article.
